# Acute Kidney Injury Related to Intoxication From Synthetic Cannabis: Don’t You Know That You’re Toxic?

**DOI:** 10.7759/cureus.23427

**Published:** 2022-03-23

**Authors:** Brett Curtis, Bishow Mahat, Michael Macklin, Jennifer Mihalo, Allie H Dakroub

**Affiliations:** 1 School of Medicine, University of Pittsburgh, Pittsburgh, USA; 2 Neurology, University of Pittsburgh Medical Center, Pittsburgh, USA; 3 Internal Medicine, University of Pittsburgh Medical Center, Pittsburgh, USA; 4 Internal Medicine/Pediatrics, University of Pittsburgh Medical Center, Pittsburgh, USA

**Keywords:** acute kidney injury, recreational drug use, young adult male, synthetic marijuana, substance recreational use

## Abstract

Acute kidney injury (AKI) occurs infrequently in young patients and often raises concern for irreversible or deadly etiologies. However, AKI related to synthetic marijuana, colloquially known as K2, is an increasingly common phenomenon in the United States and resolves quickly with fluid resuscitation. Here, we present a case of a young male who presented with severe AKI that initially raised concern for the need to start renal replacement therapy. Laboratory testing revealed an elevated osmolar gap and negative urine drug screen, while urinalysis demonstrated acanthocytes, raising concern for toxic alcohol ingestion or vasculitis. Following fluid resuscitation, his renal function improved dramatically, and he was discharged home within days of presentation.

K2-related AKI most frequently occurs in young men, mirroring the population that most frequently uses synthetic marijuana. Its exact etiology remains unknown, but direct nephrotoxicity appears to be the most plausible mechanism. No other known case has reported acanthocytes. Although objective data indicates severe illness on presentation, patients often recover rapidly to baseline and often do not suffer long-term renal impairment following conservative management.

## Introduction

Acute kidney injury (AKI) related to synthetic marijuana (K2) often presents as an acute, severe AKI most often in young male patients. Symptoms develop soon after K2 ingestion and are often gastrointestinal (GI) (e.g., nausea and vomiting) and constitutional. Multiple pathophysiologic mechanisms have been proposed, but the most plausible mechanism appears to be direct nephrotoxicity from an unidentified component of K2. Here, we report a case of AKI related to K2 ingestion that resolved rapidly with conservative management.

## Case presentation

A 29-year-old male with a history of polysubstance use, depression with a prior suicide attempt in 2019, hypertension, and gastroesophageal reflux disease presented after acute onset of malaise, nausea, vomiting, abdominal cramping, and generalized body aches. He endorsed decreased urine output over the previous few days and stated that his urine was darker and thicker than normal over this same period. He denied diarrhea, dysuria, hematuria, fevers, chills, rash, dyspnea, substance ingestion, and sick contacts.

**Figure 1:**
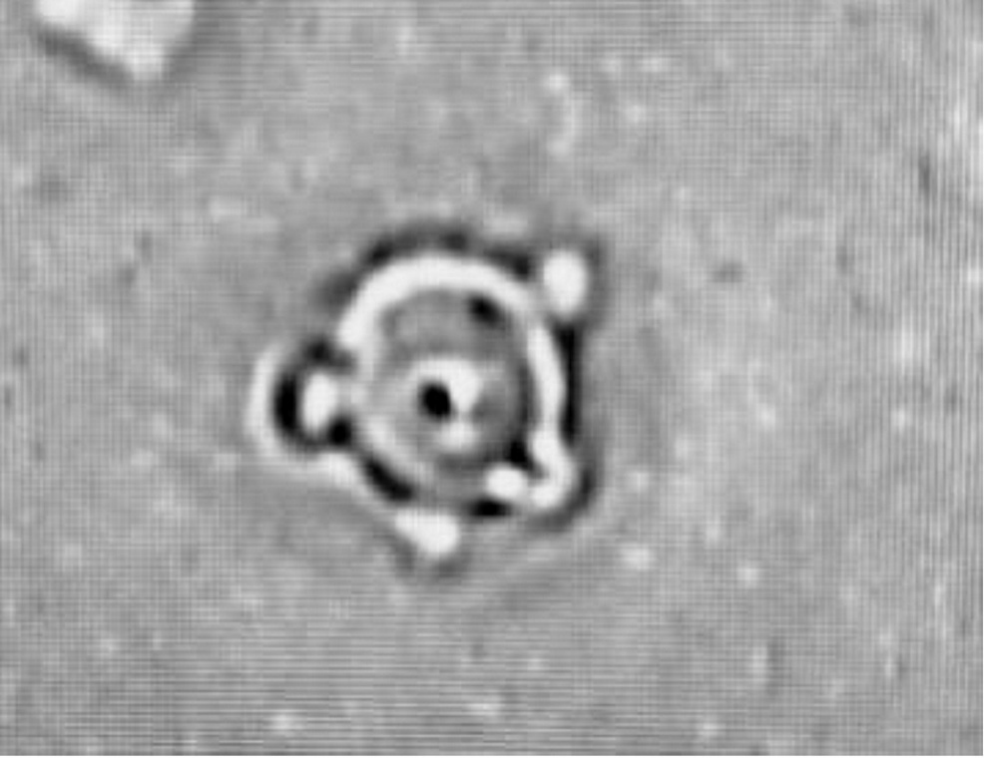
Acanthocytes, ring-shaped red blood cells with blebs of membranes on their periphery, sometimes described as red blood cells with “Mickey Mouse ears,” on UA on the first day of admission

His physical examination demonstrated diffuse abdominal and proximal muscle tenderness. Notable laboratory results on the day of hospital admission are listed in Table 1. A COVID-19 test was negative. Urinalysis demonstrated hyaline cast and 5 RBCs (Table 1). Microscopic urine analysis (US) revealed hyaline cast acanthocytes, suggesting glomerular blood loss (Figure 1). CT of the abdomen and pelvis did not find any structural lesions of the kidneys. An electrocardiogram found evidence of left ventricular hypertrophy.

**Table 1:** Notable laboratory values on the day of hospital admission

Laboratory parameters	Values
Blood urea nitrogen (BUN)	48 mg/dL
Creatinine	5.2 mg/dL
Phosphorus	9.8 mg/dL
Creatinine kinase	1,095 IU/L
White blood cells	24.5 × 10^9^/L
Serum osmolarity	305 mOsm/kg
Calculated osmolar gap	12 mOsm/kg
Anion gap	26 mEq/L
Fractional excretion of sodium (FeNa)	0.4%
Urine albumin/creatinine ratio	53.3 mg/g
Urinalysis	Acanthocytes, 5 red blood cells, 2+ blood

Throughout the first day of hospitalization, he denied ingestion of nephrotoxic agents, including alcohol, methanol, ethylene glycol, opioids, cocaine, ecstasy, or solvents. IV fluids were administered for volume resuscitation. He also received empiric fomepizole given concern for toxic alcohol ingestion on account of osmolar and anion gaps and the severity of his AKI.

On hospital day two, the patient endorsed daily use of synthetic marijuana (K2). IV fluid resuscitation was continued based on a dramatic improvement in urine output and decrease in creatinine (Cr) to 1.4 mg/dL. In the interim, an extensive workup did not reveal any significant findings. A urine toxicology screen was unremarkable. A volatile acid battery did not detect isopropanol, methanol, ethylene glycol, salicylates, tricyclic antidepressants, or acetaminophen. A vasculitis panel (e.g., antinuclear antibody, C-reactive protein, C3/4, and anti-PR3) was also negative. A renal ultrasound revealed normal-sized kidneys with no focal lesions or calculi.

On the third day of admission, the patient felt greatly improved. Renal function had normalized with a Cr of 1.1 mg/dL and an anion gap of 9 mEq/L. A planned renal biopsy was deferred given his improvement and lack of significant findings on renal ultrasound. The patient was discharged on 5 mg of lisinopril daily and outpatient follow-up with nephrology for albuminuria.

## Discussion

This case reinforces the need to maintain a broad differential for AKI while rapidly initiating appropriate treatment. In our case, we eventually identified ingestion of K2, a form of synthetic marijuana, as the likely cause of his AKI. Initially intended for use in research, synthetic cannabinoids marketed as herbal mixtures have found increasing use as recreational drugs. Its relatively low cost, perceived cannabis-like intoxication, and wide availability contribute to its growing use among young adults [1]. K2 intoxication was first reported in the United States in the early 2000s and has since increased steadily since 2012, with recent data showing almost 2,700 confirmed exposures in 2016 [2,3]. K2-related AKI has a similar history; a case series first documented this phenomenon in 2013, with an official Centers for Disease Control and Prevention warning following soon after [4,5].

In this case, our patient presented with rapid-onset AKI and elevated osmolar gap in the setting of nausea, vomiting, abdominal pain, and decreased urine output. At first, our patient’s ailment raised concern for toxic alcohol ingestion because of a dramatic rise in Cr and increased osmolar gap without any clear cause. However, his condition also held a broad differential, including rhabdomyolysis, a viral illness (e.g., influenza), vasculitis, and prerenal AKI from hypovolemia due to emesis and decreased fluid intake. While his elevated osmolar gap raised concern, the patient’s gap of 12 mOsm/L was nonspecific, and moieties associated with K2 are not known to be osmotically active; only a gap greater than 20 mOsm/L most supports toxic alcohol ingestion [1,6]. We initially suspected rhabdomyolysis related to a viral infection based on his elevated CK and diffuse myalgias. However, AKI from rhabdomyolysis often does not occur until much higher CK levels, and the patient lacked other features of viral illnesses, such as a high fever or respiratory symptoms [7]. Acanthocytes discovered on urinalysis indicated a glomerular source of bleeding potentially secondary to vasculitis, but this was refuted with an otherwise unremarkable workup. K2 ingestion was not initially on our differential based on the patient’s unclear toxin exposure history. However, he later endorsed daily K2 ingestion, and his symptoms greatly improved with IV fluid resuscitation.

AKI with hospitalization remains a rare event among people aged 22-39, with an incidence of less than one case per 1,000 patient-years [8]. Our case’s severe initial findings appeared concerning rapid progression to renal replacement therapy or hemodialysis. Thankfully, his condition resolved within days with supportive treatment. To our knowledge, no other case has been reported with acanthocytes on urinalysis. More importantly, acanthocytes strongly suggest glomerular damage, a different mechanism from prior reports that have suggested this to be a tubular process [9].

Although K2 use has increased over the past decade, large regional disparities in K2 intoxication are reported and may even vary on a quarterly basis throughout the United States [10]. K2’s toxicities may also include cardiac events, generalized tonic-clonic seizures, delirium, and other neurological and psychiatric manifestations [11]. Patients suffering from K2 intoxication match the demographics presented in this case; they are typically males in their early 20s presenting with nausea, vomiting, and diffuse abdominal pain with possible oliguria [12]. Surveys confirm that this demographic matches that of the average K2 user [13]. However, more detailed data on K2’s epidemiology (e.g., racial or ethnic demographics of K2 users) and effects on healthcare systems, such as hospitalizations and ICU admissions, is not known. Workup in K2-related AKI is typically unremarkable (e.g., autoimmune and infectious), and renal function may recover spontaneously or with supportive treatment [9].

Although the clinical pattern of K2-related AKI appears relatively well-understood, its pathogenesis remains less so. The proposed causes of K2-related nephrotoxicity include rhabdomyolysis, hypovolemia, and toxin exposure. Some case reports of K2 ingestion have shown rhabdomyolysis or elevated CK in the setting of AKI [14,15]. As with our patient, these levels are not high enough to cause nephrotoxicity. Other cases have reported only mild elevations in CK with no urine myoglobin [4,9]. Azotemia from volume depletion is supported by a rapid resolution of symptoms following fluid resuscitation and renal biopsy findings of acute tubular necrosis (ATN) in other reported cases. However, this relationship is correlational, not causational, and acute interstitial nephritis has been reported on biopsy without an otherwise identifiable pharmacologic agent [15]. Finally, the cause of the patient’s gastrointestinal symptoms remains unclear. Several mechanisms, including the action of K2 on synthetic cannabinoid receptors in the GI tract, possible adulterants in the ingested K2, and localized vasospasm, are possible but have not been definitively confirmed [16].

Nephrotoxicity stands as the most plausible cause of K2-related AKI. However, K2 is not a defined moiety. While some agents, such as XLR-11, have been implicated [9], synthetic cannabinoids represent a diverse class of drugs with new moieties continually being identified [11]. The nonstandard manufacturing process of synthetic cannabis leads to heterogeneous compounds and drug concentrations. This makes identifying the causative agent and understanding the mechanism of nephrotoxicity related to K2 difficult. Preclinical data further muddies this issue. K2 and other synthetic cannabinoids, strong agonists of cannabinoid (CB) receptors, may concentrate in kidneys because CB1 and CB2 receptors are expressed by podocytes within human glomeruli [17]. As a result, some have implicated CB2 activation in the kidneys by K2 in the nephrotoxic effects of K2 [13]. However, animal studies suggest that the activation of these receptors may actually have a protective effect against renal degeneration [18]. Regardless, renal biopsies and clinical reports have shown that ATN remains the most common pathology, supporting nephrotoxicity as the mechanism for kidney injury from K2 [9].

Regardless of the mechanism, the management of K2-related AKI is often uncomplicated. Treatment remains supportive with IV fluids and monitoring of renal function and only occasionally requires renal replacement therapy [9]. Patients often recover spontaneously with or without treatment. K2-related AKI may not inflict significant mortality; for example, a recent case series of K2-related critical illness showed that only one of 30 patients admitted to ICUs over a two-year period in NYC died, suggesting a very favorable prognosis for even the most severe K2 intoxications [19]. The long-term prognosis is likely less favorable. The most relevant data comes from a meta-analysis suggesting that individuals with any history of AKI have a much higher risk of developing chronic kidney disease and end-stage renal disease when compared with those without any AKI history [20]. Outpatient follow-up and management (e.g., addiction medicine referral) are necessary to decrease the chance of repeat intoxication, co-ingestion with other substances, and other psychosocial concerns.

## Conclusions

K2-induced AKI is a rare cause of acute prerenal AKI with an unclear mechanism and severe objective findings that have been increasingly reported over the past decade. The average patient demographic is likely young men with a history of recreationally using K2. Patients often recover their baseline renal function spontaneously or with supportive care and rarely require renal replacement therapy. Clinicians should maintain suspicion for K2-induced AKI when otherwise healthy young patients with negative drug screens present with severe, acute prerenal AKI.

## References

[1] Castaneto MS, Gorelick DA, Desrosiers NA, Hartman RL, Pirard S, Huestis MA (2014). Synthetic cannabinoids: epidemiology, pharmacodynamics, and clinical implications. Drug Alcohol Depend.

[2] Riederer AM, Campleman SL, Carlson RG, Boyer EW, Manini AF, Wax PM, Brent JA (2016). Acute poisonings from synthetic cannabinoids - 50 U.S. Toxicology Investigators Consortium Registry sites, 2010-2015. MMWR Morb Mortal Wkly Rep.

[3] (2021). National Institute on Drug Abuse (NIDA): Synthetic cannabinoids (K2/spice) unpredictable danger. https://www.drugabuse.gov/drug-topics/trends-statistics/infographics/synthetic-cannabinoids-k2spice-unpredictable-danger.

[4] Bhanushali GK, Jain G, Fatima H, Leisch LJ, Thornley-Brown D (2013). AKI associated with synthetic cannabinoids: a case series. Clin J Am Soc Nephrol.

[5] (2013). Acute kidney injury associated with synthetic cannabinoid use--multiple states, 2012. MMWR Morb Mortal Wkly Rep.

[6] Kraut JA, Kurtz I (2008). Toxic alcohol ingestions: clinical features, diagnosis, and management. Clin J Am Soc Nephrol.

[7] Latham J, Campbell D, Nichols W, Mott T (2008). Clinical inquiries. How much can exercise raise creatine kinase level--and does it matter?. J Fam Pract.

[8] United States Renal Data System (2018). Chapter 5: Acute kidney injury. 2018 USRDS annual data report: Epidemiology of kidney disease in the United States.

[9] Srisung W, Jamal F, Prabhakar S (2015). Synthetic cannabinoids and acute kidney injury. Proc (Bayl Univ Med Cent).

[10] Roehler DR, Hoots BE, Vivolo-Kantor AM (2020). Regional trends in suspected synthetic cannabinoid exposure from January 2016 to September 2019 in the United States. Drug Alcohol Depend.

[11] Luethi D, Liechti ME (2020). Designer drugs: mechanism of action and adverse effects. Arch Toxicol.

[12] Hoyte CO, Jacob J, Monte AA, Al-Jumaan M, Bronstein AC, Heard KJ (2012). A characterization of synthetic cannabinoid exposures reported to the National Poison Data System in 2010. Ann Emerg Med.

[13] Vandrey R, Dunn KE, Fry JA, Girling ER (2012). A survey study to characterize use of Spice products (synthetic cannabinoids). Drug Alcohol Depend.

[14] Adedinsewo DA, Odewole O, Todd T (2016). Acute rhabdomyolysis following synthetic cannabinoid ingestion. N Am J Med Sci.

[15] Zarifi C, Vyas S (2017). Spice-y kidney failure: a case report and systematic review of acute kidney injury attributable to the use of synthetic cannabis. Perm J.

[16] Hakimian D, Benson AA, Khoury T (2021). Gastrointestinal manifestations of synthetic cannabinoids: a retrospective cohort study. BMC Gastroenterol.

[17] Barutta F, Corbelli A, Mastrocola R (2010). Cannabinoid receptor 1 blockade ameliorates albuminuria in experimental diabetic nephropathy. Diabetes.

[18] Nam DH, Lee MH, Kim JE (2012). Blockade of cannabinoid receptor 1 improves insulin resistance, lipid metabolism, and diabetic nephropathy in db/db mice. Endocrinology.

[19] Kourouni I, Mourad B, Khouli H, Shapiro JM, Mathew JP (2020). Critical illness secondary to synthetic cannabinoid ingestion. JAMA Netw Open.

[20] Coca SG, Singanamala S, Parikh CR (2012). Chronic kidney disease after acute kidney injury: a systematic review and meta-analysis. Kidney Int.

